# Editorial: Methods in structural biology: Cryo-EM

**DOI:** 10.3389/fmolb.2022.1041373

**Published:** 2022-10-24

**Authors:** Olga S. Sokolova

**Affiliations:** Department of Bioengineering, Faculty of Biology, Moscow Lomonosov University, Moscow, Russia

**Keywords:** cryo-EM, image analysis, electron radiation damage, neural network, shared facilities

## Introduction

In the 20th century, structural biology has succeeded in obtaining enormous amounts of information leading to spectacular results in X-ray crystallography. Knowledge of the structure of macromolecules provides for interpreting conformational changes in molecular machinery upon activation or inhibition. By 2022, more than 195,000 structures of proteins, including large molecular machines, have been deposited to the pdb.org database. Following genomics, proteomics, transcriptomics, and metabolomics, a branch of science emerged called “structural proteomics.” It is interesting to mention that from 1923 to 2021, 75 Nobel prizes have been awarded to structural biologists (31 prizes in chemistry and 44 in physiology and medicine).

In the second decade of the 21st century, we witnessed an unprecedented breakthrough in the area of cryoelectron microscopy (cryo-EM). This was partially a result of a long-awaited 2017 Nobel prize in chemistry, which was awarded to Jacques Dubochet, Joachim Frank, and Richard Henderson for their impressive research in the development of the cryo-EM approach. On the other hand, advances in the improvement of cryoelectron microscopes, field emission cathodes, and modern electron-optical systems led to the improvement of electron beam characteristics.At the beginning of the 21st century, direct detection devices (DDD) with decreased signal-to-noise ratio were created which allowed for motion correction (McMullan et al., 2014). Finally, years of work spent on fully automating data acquisition and processing have led to the creation of platforms that now make efficient use of tools and computational resources, making cryo-EM of macromolecules available to the broader scientific community. Recent technical developments have made cryo-EM the primary method of structural biology, especially for characterizing complex samples that resist crystallization, can only be obtained in small quantities, or suffer from compositional or conformational heterogeneity. This has been impressively demonstrated by the determination of the *de novo* structure of the TRPV channel (Liao, et al., 2013) at 3.4 Å resolution, the structure of β-galactosidase at 2.2 Å resolution (Bartesaghi et al., 2015), and the structure of glutamate dehydrogenase at 1.8 Å resolution (Merk et al., 2016), and, finally, the structures of apoferritin at atomic resolution (Nakane et al., 2020; Yip et al., 2020), which indicates a new era of cryo-EM.

Given the wealth of recent advances in cryo-EM, there is clearly a need for putting together a special Research Topic that would include up-to-date Reviews and Original Research describing novel structural methods in Cryo-EM.

We started with the paper (doi.org/10.3389/fmolb.2022.937253) that highlights the role of sample preparation. It still remains a major challenge for high-resolution structural cryo-EM studies. In the last years, the use of monolayer graphene grids was increased to minimize the background and to reveal near-atomic structures of proteins with a small molecular weight and low concentration (Patel et al., 2021).

Next, the review of Shi and Huang (10.3389/fmolb. 2022.988928) underlines an important point: electron radiation damage to macromolecules is one of the major resolution limiting factors in cryo-EM. The authors compared two techniques: single particle analysis (SPA) and micro-crystal electron diffraction (MicroED) and revealed that the minimum dose of electron irradiation for SPA is 10 times higher than that reported by MicroED. Indeed, for multilayered crystals, the probability of the inelastic scattering of electrons by a macromolecule increases proportionally to the number of crystal layers. The reviewed data will benefit all applications of cryo-EM, especially cellular structure analysis by tomography.

In this regard, the review of Navarro (doi.org/10.3389/fmolb.2022.934465) underlines the method of quantitative tomography. The author discusses the impact of sample thickness, which influences all aspects of the cryo-ET workflow. Also, the electron dose and sample preparation, including CryoFIB or Cryo-Lift-Out, were reviewed. This review yielded the maximum number of views among all papers submitted to our Research topic and has more downloads than 20% of all Frontiers articles.

We are glad that our Research Topic motivated the submission of original articles. Schöenfeld et al. (doi.org/10.3389/fmolb.2022.919994) presented GPU/ISAC: a 2D clustering algorithm that provides high-quality class averages on a single GPU machine. Hamitouche and Jonic (doi.org/10.3389/fmolb.2022.965645) reported a novel neuronal network, DeepHEMNMA, which is based on a deep learning approach. Authors noted that the network should be trained for each molecular complex, depending on the shape of the complex. Nevertheless, the described network works faster than previous tools developed by this research group.

Importantly, the article of Walsh et al. (10.3389/fmolb. 2022.960940) describes the Harvard Cryo-Electron Microscopy Center for Structural Biology, which was formed as a consortium between several leading research institutions in the Greater Boston area. Since maintaining a full-featured cryo-EM center requires large expenses, it is necessary to combine efforts to optimize research productivity, while training users to be expert electron microscopists. In this article, the authors describe their established pipelines.

**FIGURE 1:**
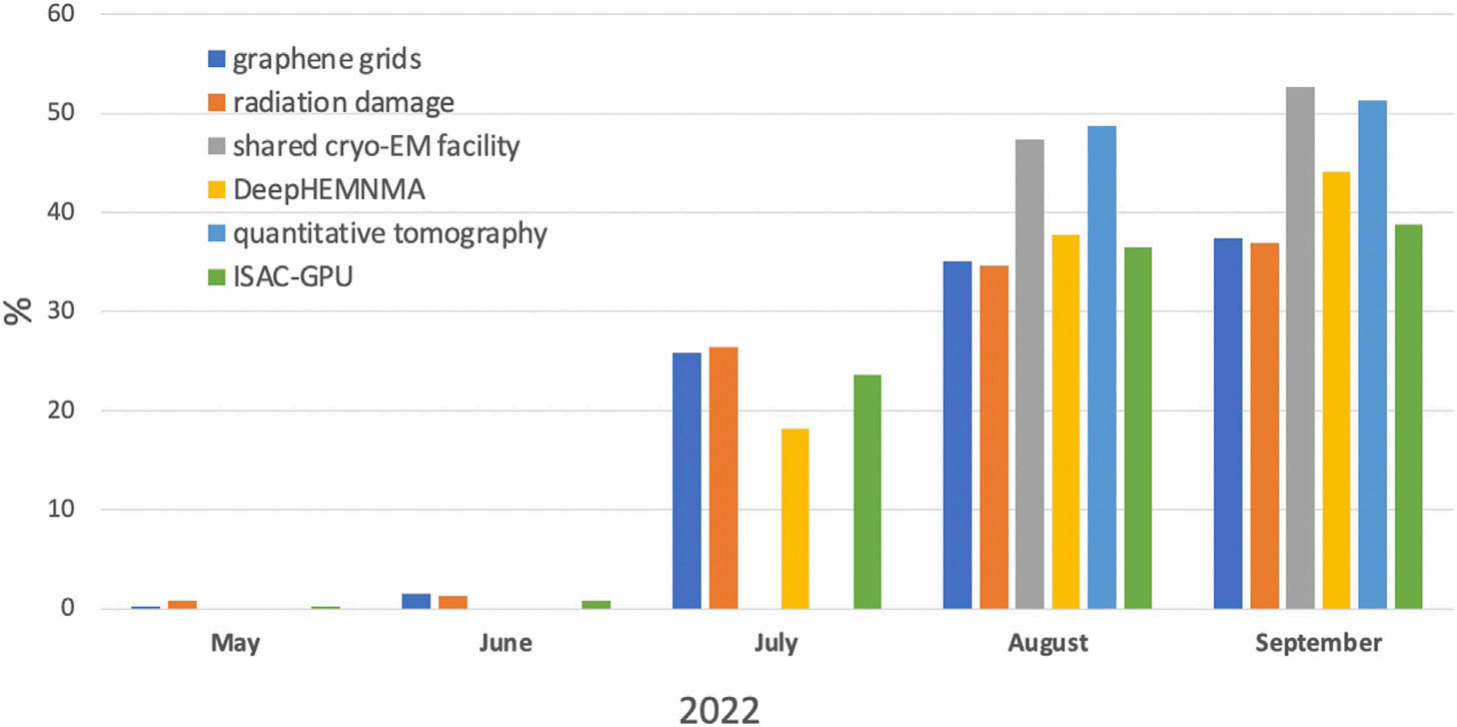
Views of papers, accepted to the Research Topic Methods in Structural Biology: Cryo-EM, according to Frontiers impact score per month in % to the total number of views.

All articles from the Structural methods in Cryo-EM research topic attracted the attention of the research community: in the last 5 months of 2022, all papers together yielded more than 10,000 views according to the Frontiers impact score (Figure 1). From May to July there were few hits, as the articles were undergoing the review process. As expected, in the last 2 months, Methods papers attracted more attention.
